# Thermo-Ablative Fractional CO_2_ Lasers Combined with 1540 nm Wavelengths Is a Promising Treatment Option in Stress Urinary Incontinence

**DOI:** 10.3390/medsci13010025

**Published:** 2025-03-01

**Authors:** Maurizio Filippini, Sara Elmi, Jessica Sozzi, Laura Pieri, Irene Fusco, Tiziano Zingoni, Pablo González-Isaza

**Affiliations:** 1 Department of Obstetrics and Gynaecology, Hospital State of Republic of San Marino, 47893 San Marino, Italy; mfilippini@omniway.sm (M.F.); sozzi.jessica@hotmail.com (J.S.); 2 El.En. Group, 50041 Calenzano, Italy; famas@elen.it (S.E.); l.pieri@deka.it (L.P.); t.zingoni@elen.it (T.Z.); 3 Ginecología y Obstetricia, Centro Médico Teknon, 08017 Barcelona, Spain; pagonza@hotmail.com

**Keywords:** stress urinary incontinence, laser therapy, CO_2_ ablative laser, 1540 nm non-ablative laser, combination laser treatment

## Abstract

**Background/Objectives**: Stress urinary incontinence (SUI) is a common and often under-reported condition that significantly impacts quality of life. SUI is more than just a physical issue; it can also affect social interactions, mental health, and emotional well-being due to the embarrassment and limitations it can cause. SUI is often acquired during pregnancy and childbirth as a result of pelvic floor muscle weakness. The aim of this study was to evaluate the effectiveness of an innovative dual-wavelength laser system (CO_2_ + 1540 nm) in SUI management. **Methods**: A total of 56 women affected by SUI were enrolled in this study. Half of the patients were treated with CO_2_ alone, while the other half were treated with the combination of CO_2_ + 1540 nm wavelengths. The patients were split into four groups based on the type of treatment they received and their menopausal status. Data were acquired at baseline and at various follow-ups (T1, T2, and T3, respectively, after the first, second, and third treatment). The Visual Analog Scale (VAS) (score 0–10) was used. Cystoscopic images were acquired before and at the end of the laser treatment cycle. **Results**: At the end of the treatment, the patients in each group were very satisfied, on average. In each group, the treatment led to a statistically significant improvement in the SUI VAS score between baseline and follow-up after the first treatment; in both groups 3 and 4, the treatment led to a significant change in the dryness score, both from baseline to T1 (*p* < 0.05) and also for T2 and T3 compared to baseline. Finally, cystoscopic photos showed an evident increase in mucosa epithelial thickness after the laser treatment cycle. **Conclusions**: The use of a dual-wavelength laser system (CO_2_ + 1540 nm) was proven to be well tolerated and safe, with promising outcomes in reducing SUI symptoms, especially in non-menopausal patients.

## 1. Introduction

Stress urinary incontinence (SUI), which is defined as the involuntary leakage of urine, is a critical public health issue with a significant social outcome. SUI affects millions of women globally and is estimated to afflict 4–35% of adult women [1]. SUI is often acquired during pregnancy and childbirth as a result of pelvic floor muscle weakness that causes the urethra to be maintained against the anterior vaginal wall, as well as urethral sphincter dysfunction, which is responsible for intrinsic and extrinsic continence [2]. A weaker urogenital tract results from hormone deprivation, which inhibits collagen synthesis and depletes collagen reserves, causing the periurethral tissue to relax [3].

Urinary incontinence treatment options mostly include “conservative therapy” (which avoids intrusive procedures), medication, and surgery. The conservative management of stress urinary incontinence should be offered first, and this can include the training of the pelvic floor muscles and behavioral therapy with lifestyle changes such as weight loss, bladder training, pharmacotherapy, and the use of mechanical devices, vaginal cones, and electrical stimulation. These are often offered by nurses or physiotherapists with specialized training. Patients are frequently given a combination of these non-invasive treatments [4]. These treatments necessitate a high level of patient compliance; for this reason, surgery is still a more appealing and practical option for treating SUI, even though it is more intrusive and involves difficulties and a longer recovery time [5,6].

Surgery is part of the current treatment conducted for SUI to restore adequate urethral resistance and stop urine leaking during periods of elevated intra-abdominal pressure [7]. Traditional methods involve the use of plication sites, which results in cicatrization and the formation of collagen. Polypropylene tapes are currently used to make neoligaments, which employ a foreign-body response to produce collagen.

According to the literature, surgical procedures can result in severe and adverse events in 2% to 12% of cases [5]; however, advances in science and technology have resulted in an improvement in clinical outcomes when less invasive procedures are used, so this type of treatment is only used when conservative measures fail [8]. In this regard, current research supports laser therapy as an alternate and effective option for SUI. Transvaginal laser therapy has been investigated recently as a less invasive and successful approach to treating SUI and genito-urinary syndrome of menopause (GSM) [9,10,11,12]. Laser therapy for SUI has been adopted because it may enhance support structures, thus guaranteeing that the tension created by the active contraction of the pelvic floor muscles (PFMs) can be used for successful non-surgical treatment for female SUI and other conditions brought on by decreased pelvic floor support [13], thus having a positive impact on the prolapse of pelvic organs (POP) [14]. Laser treatment is a good, painless outpatient procedure with no side effects in post-menopausal women with GSM, as evidenced by the literature, which shows that there are extensive beneficial effects of laser therapy (significant improvement in dyspareunia, dryness, burning, itching, dysuria, urgency, and urinary symptoms, as well as decreased UI scores) [10,15,16].

Transvaginal CO_2_ laser treatment decreased UI symptoms in women with vaginal atrophy, according to Casiraghi et al. [17]. Thermo-ablative treatments, such as the use of fractional CO_2_ lasers, involve applying controlled heat to the vaginal and pelvic tissues. The heat creates microscopic thermal injuries in the tissues, which then stimulates the body’s natural healing response. This leads to increased collagen production and tissue remodeling, thus strengthening the vaginal and pelvic floor tissues. Appropriate collagen synthesis would help to relieve strain in the pubo-cervical fascia and restore ligament function. Through a natural mechanism of cellular self-regeneration, the CO_2_ laser fractionated through thermal shock proteins promotes collagenogenesis at the pubo-cervical fascia level by activating fibroblasts and forming an autologous mesh, which reduces the hypermotility of this dysfunction without the potential complications associated with mesh use. Laser treatments also stimulate the production of elastin, which helps restore the elasticity of the vaginal and pelvic tissues. This is particularly important for post-menopausal women, as the lack of estrogen leads to the thinning and dryness of the vaginal walls. This restoration can significantly reduce symptoms like vaginal dryness, burning, and itching, which are common in GSM [16]. Laser therapy is generally well tolerated, with minimal downtime. Women typically experience only mild discomfort, which resolves shortly after the laser therapy session. There are no major side effects in most cases, thus making this therapy a favorable option for many women, especially those who wish to avoid more invasive treatments. In clinical studies, laser therapy has been associated with a low risk of complications, and women report high satisfaction rates with improvements of up to 78% [4,12,18].

Several studies on dermatological applications show that combining CO_2_ with infrared wavelengths enhances the thermal impact on the vaginal and pelvic tissues; this results in more effective tissue remodeling therapies, including faster healing periods, increased cell turnover, and deeper stimulation. This dual-wavelength technology combines the ablative features of CO_2_ lasers with profound non-ablative and non-coagulative qualities to maximize its effectiveness while minimizing adverse effects [19,20,21].

The 1540 nm wavelength, which has a non-ablative thermal penetration depth of around 3 mm, affects cultured fibroblasts by decreasing matrix protein production and increasing collagen-related gene expression [22,23]; this process includes genes involved in the synthesis of collagen type I, collagen type III, and fibronectin, all of which contribute to the tissue’s strength and elasticity. Laser treatment can also help modulate matrix protein production. While collagen production increases, the laser can reduce the production of some other matrix proteins that may contribute to fibrosis or scarring, such as excessive amounts of collagen type I or III that could lead to the stiffening of tissues. This balance helps in collagen remodeling, resulting in stronger yet more flexible tissues, which improves the elasticity of the vaginal and pelvic tissues [22,23]. In particular, Magni and colleagues showed that there was a rise in the mitochondrial electrical potentiality’s cellular activity and a notable improvement in type III collagen expression, indicating the effectiveness of laser-induced neocollagenesis [24].

To evaluate effectiveness during SUI management and ascertain whether CO_2_ laser therapy is different from combination laser treatment (CO_2_ and 1540 nm), an innovative dual-wavelength laser system was utilized.

## 2. Materials and Methods

The research conducted consisted of a retrospective, monocentric analysis involving all women who underwent evaluation for SUI at the Institute for Social Security of the Republic of San Marino. In view of this, we examined 56 women affected by SUI; half of them were already in menopause, and the other half were not. The patients were split up into four groups (see Table 1). Exclusion criteria were the following: ongoing pregnancy, breastfeeding, or having neoplasms at the time of treatment.

**Figure 1:**
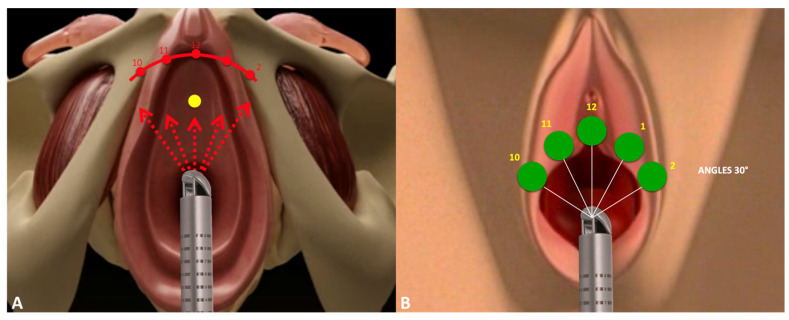
A graphic illustration of the laser technique. A 90° vaginal laser-emitting probe was inserted up to the bladder neck to transmit thermal energy with laser spots in five different suburethral areas (**A**), corresponding to the 10, 11, 12, 1, and 2 h of the clock and with angles of 30° (**B**).

**Figure 2:**
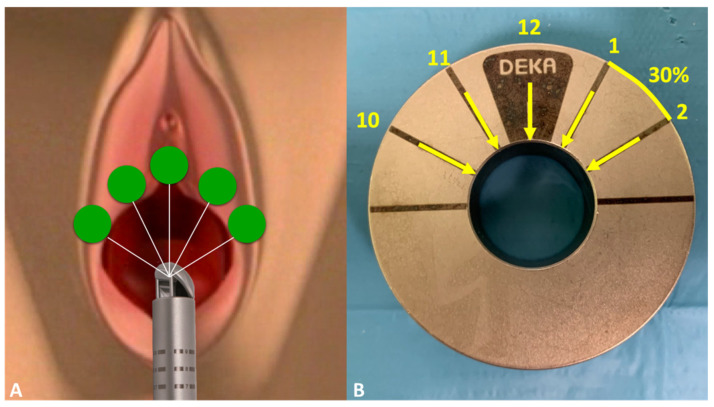
Laser spots can be applied freehand (**A**) or through a specific ring that reflects the technique’s established repeat points (**B**).

**Figure 3:**
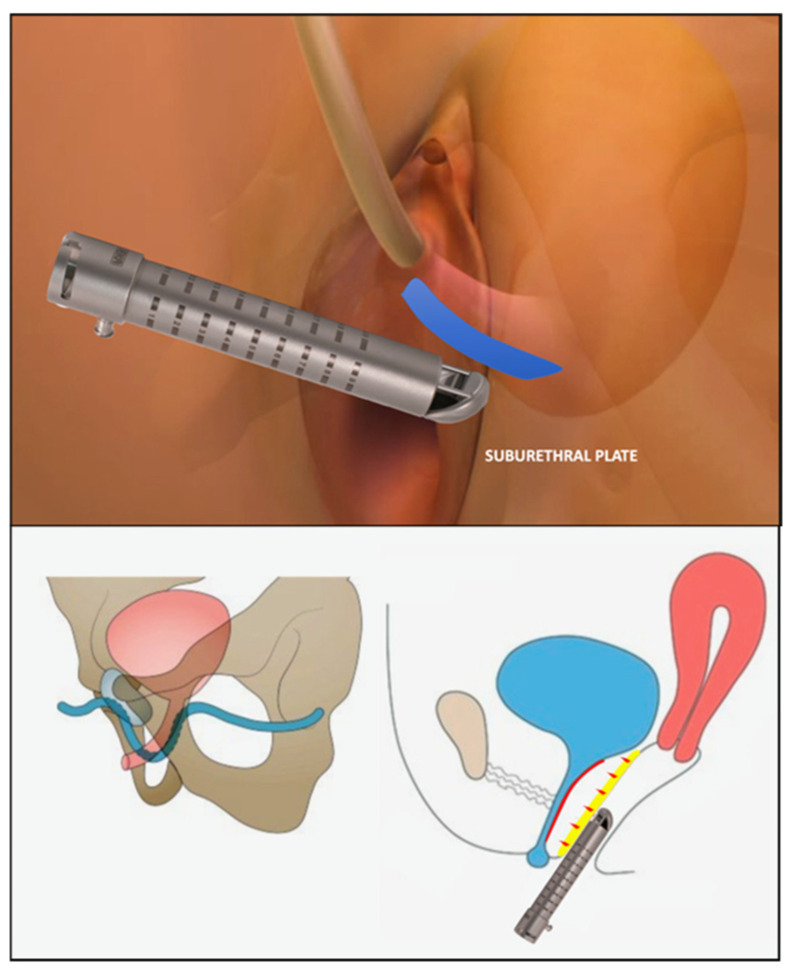
Graphic illustration of mechanism of action of laser technique.

All the participants underwent transvaginal laser treatments with the DuoGlide system (DEKA M.e.l.a, Florence, Italy), with the following settings applied: a power of 40 W, a dwell time of 2000 ms, a DOT spacing of 1000 mm, a SmartStack parameter of 4, and double-pulse mode. A 90° vaginal laser-emitting probe was then inserted up to the bladder neck, targeting the sub-point through five steps with 30° degrees of thermal energy, and then rotated and removed for the entire front length of the vagina (Figure 1A,B). Laser spots can be applied freehand or through a specific ring that reflects the technique’s established repeat points (Figure 2). This technique’s goal is to reproduce a support plate under the urethra through the photothermal effect on the vaginal wall triggered by the thermal energy of the laser, thus mimicking the mechanism of action of suburethral slings. Indeed, the high temperatures generated by laser action can denature the collagen triple helix’s highly ordered structure, thus leading to the contraction of collagen into thicker and shorter fibers and consequently to the induction of neocollagenesis. Collagen strengthening enhances the thickness, flexibility, and compactness of the vaginal wall, thus resulting in better suburethral support and urine continence. The mechanism of action is illustrated in Figure 3.

In addition to CO_2_ therapy, the participants undergoing combination therapy received the 1540 nm treatment with an energy of 200 mJ (power of 5 W, dwell time of 10 ms), using the same handpiece. To ensure sufficient tissue healing and response assessment, patients underwent three therapy sessions, separated by 8–12 weeks.

Before beginning therapy, participants provided signed informed consent forms, in accordance with the Declaration of Helsinki. Before analysis, records/information were anonymized and de-identified. The data at baseline (before treatment) and at various follow-ups (T1, T2, and T3, respectively, after the first, second, and third treatment) were processed using SPSS 21.0 (SPSS, Chicago, IL, USA).

The patients were initially compared for their age and number of vaginal births using a Student’s *t*-test analysis. The subjective primary outcome measure was the use of a Visual Analog Scale (VAS) (score 0–10) to gauge treatment satisfaction, which allowed for a quick and easy assessment of the symptoms and the effects of urinary incontinence on the study population’s quality of life [10]. The VAS was also used to assess SUI symptoms and vaginal dryness, with scores ranging from 0 to 5 and from 0 to 10, respectively.

Finally, cystoscopic images were acquired before and at the end of the laser treatment cycle.

A comparison between symptom ratings within the same group was performed using the Wilcoxon test, while a comparison between groups was conducted using the Mann–Whitney test. Any analyses with a *p*-value of <0.05 were considered statistically significant.

## 3. Results

**Figure 4:**
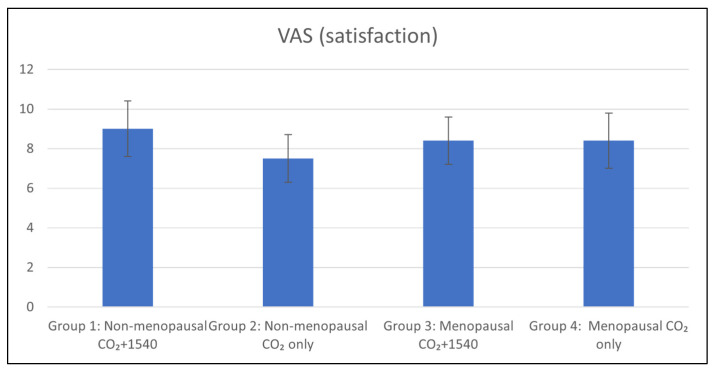
Visual Analog Scale (VAS) satisfaction of four groups. Non-menopausal women treated with CO_2_ + 1540 nm (Group 1) reported considerably higher levels of satisfaction than those treated with CO_2_ only (Group 2).

At the end of the treatment, the patients in each group were very satisfied, on average, and had the following VAS scores: 9.0 ± 1.4 for Group 1, 7.5 ± 1.2 for Group 2, 8.4 ± 1.2 for Group 3, and 8.4 ± 1.4 for Group 4. Among the non-menopausal patients (Groups 1 and 2), the satisfaction in Group 1 was significantly higher than that in Group 2 (Figure 4).

**Figure 5:**
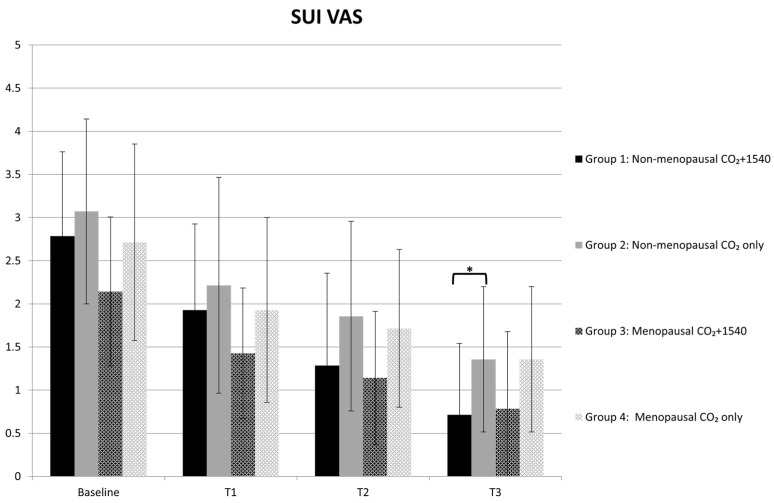
The SUI VAS * in non-menopausal patients. A significant improvement in the T3 VAS score was observed in Group 1 compared to Group 2.

In each group, the treatment led to a statistically significant improvement in the SUI VAS score between baseline and follow-up after the first treatment; the improvement was statistically significant between all the final SUI VAS scores (T3) and the respective baseline of each group. Furthermore, in non-menopausal patients, a significant improvement in the T3 SUI VAS score was observed in Group 1 compared to Group 2 (Figure 5).

Vaginal dryness was evaluated only in women who also reported this symptom, in addition to SUI, and only in the post-menopausal patients (groups 3 and 4). In both groups, the treatment led to a significant change in the dryness score, both from baseline to T1 (*p* < 0.05) and also for T2 and T3 compared to baseline. There were no significant differences between the two groups either at baseline or at T3 (Table 2).

**Figure 6:**
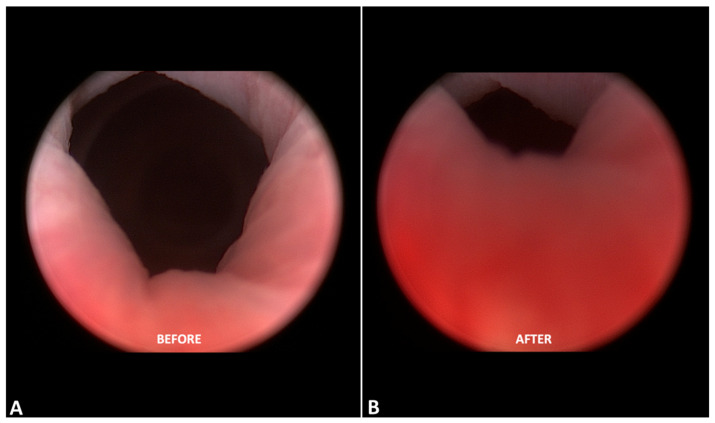
Cystoscopic images of patient of Group 3 before (**A**) and after laser treatment cycle (**B**). Evident increase in epithelial thickness after end of laser treatment cycle was observed.

Finally, cystoscopic photos (Figure 6) showed an evident increase in mucosa epithelial thickness after the laser treatment cycle, providing further confirmation of the improvement in stress urinary incontinence.

## 4. Discussion

The present study indicates that the use of CO_2_ lasers with and without the use of 1540 nm wavelengths led to improvements in SUI (reduction in urinary leakage, improved bladder control, enhanced tissue strength and elasticity, and better overall quality of life) in both menopausal and non-menopausal patients, as well as improvements in vaginal dryness in menopausal patients, even though the dual-wavelength treatment provided more benefits in non-menopausal women. Notably, the patients expressed very high levels of satisfaction with the procedure and reported significant improvements in their quality of life. It is important to highlight that no adverse events were documented during this study in any of the four groups.

By generating thermal alterations in tissues, CO_2_ lasers can trigger an inflammatory healing response, leading to various histological, cytological, metabolic, and gene expression modifications that can repair and reshape tissues by stimulating collagen production and the structural reorganization of collagen fibers [9,25,26]. CO_2_ lasers have a major impact on collagen dynamics because they not only boost fibroblast proliferation but also regulate the production of critical factors involved in collagen synthesis. These lasers stimulate the production of basic fibroblast growth factor (bFGF), which decreases collagen synthesis while inhibiting the production of transforming growth factor-beta1 (TGF-β1), which promotes collagen synthesis. This dual action promotes a balanced collagen structure, preventing excessive fibrosis and abnormal wound healing [27]. This control of collagen production and structure is critical for effective wound healing and fibrosis reduction.

The photobiomodulation impact of a 1540 nm wavelength therapy on the proliferation of cultured fibroblasts and their capacity to produce type I and III collagen was assessed in a recently published study [24], with encouraging findings. Due to their unique spatial shape, 1540 nm wavelengths enable the use of non-ablative heat that is uniform and continuous across the scan region, reaching great depths in the tissues that are not easily accessible with ablative lasers alone without raising CO_2_ energy.

A plausible pathogenetic theory for the improvement in SUI in menopausal women following laser treatment could be attributed to alterations in the atrophic urethral epithelium at the intrinsic sphincter level. In our investigation, this theory was validated using a cystoscopic assessment that showed that there was an evident increase in mucosa epithelial thickness after the laser treatment cycle, highlighting a similarity with the use of bulky systems, as the increase in epithelial thickness produced by the thermal energy delivered induces histological changes, leading to the partial or total narrowing of the urethral orifice at the sphincter level.

SUI in post-menopausal women is primarily caused by a reduction in estrogen levels, which leads to several changes in the urinary tract and pelvic floor. These include the loss of muscle tone, vaginal atrophy, changes in the urethral mucosa, and alterations in collagen, which result in weakened structural support and increased urethral mobility. In non-menopausal patients, SUI is caused by urethral hypermobility.

In post-menopausal patients with SUI or in non-menopausal patients with urethral hypermobility, the CO_2_ laser treatment applied to the vaginal area, targeting the subpubic area, may enhance the collagen content and structure of the pubo-cervical fascia, as well as increasing mucosal thickness, thereby strengthening the support of the urethra and bladder neck [28,29].

The use of the second wavelength (1540 nm) does not result in significant differences compared to the use of a CO_2_ laser alone in post-menopausal patients, while it provides a significant advantage in younger, non-menopausal patients. The combination of CO_2_ and 1540 emissions resulted in an increase in deep non-ablative heating, without enhancing the ablative effects of the CO_2_ laser alone [30], offering greater benefits for younger patients, in whom SUI is caused by urethral hypermobility. Selecting the right wavelengths and emitting them sequentially on a single energy pulse (DOT) improves tissue effects, reduces energy dosage, and improves safety, resulting in less downtime after treatment. This could explain the greater satisfaction of non-menopausal patients in Group 1 compared to Group 2.

### Study Limitations

This study has some limitations. The absence of a control group is undoubtedly a drawback, as it means that this study could not directly compare the experimental group (those receiving the treatment or intervention) to the group that did not receive the treatment. It would be interesting to extend the analysis to a bigger group of patients. Moreover, we will work to plan a future trial that includes both a control group and more patients.

## 5. Conclusions

The use of CO_2_ alone and combined with the 1540 nm wavelength is well tolerated and safe and has shown promising results in terms of relieving SUI symptoms in both post-menopausal and non-post-menopausal patients. Specifically, the use of the second wavelength in the combined CO_2_-1540 nm treatment was found to be more beneficial in non-post-menopausal patients due to the deeper and prolonged effect induced by the 1540 nm wavelength compared to CO_2_ alone. Larger samples and further studies are needed, such as randomized trials with a control group, to confirm the efficacy and advantages of laser technology in SUI therapy.

## Figures and Tables

**Table 1:** The study population is divided into 4 groups depending on age (menopausal or not) and treatment type (CO_2_ alone or CO_2_ + 1540 nm).

	N°/Type of Patients	Age (Mean ± Devst)	Spontaneous Births (Mean ± Devst)	Laser Treatment
Group 1	14 non-menopausal women	47.8 ± 5.3	1.5 ± 1.1	Combined laser treatment (CO_2_ and 1540 nm)
Group 2	14 non-menopausal women	47.7 ± 5.2	1.7 ± 0.9	CO_2_ laser treatment
Group 3	14 post-menopausal women	58.6 ± 6.1	1.0 ± 1.1	Combined laser treatment (CO_2_ and 1540 nm)
Group 4	14 post-menopausal women	58.5 ± 6.1	1.5 ± 0.9	CO_2_ laser treatment

**Table 2:** VAS scores in terms of vaginal dryness.

	Group 3 (n = 12)	Group 4 (n = 11)
	Mean ± Devst	Mean ± Devst
Baseline	7.3 ± 1.7	8.3 ± 1.5
T1	5.8 ± 1.9	6.4 ± 1.6
T2	4.8 ± 2.1	4.9 ± 2.8
T3	3.7 ± 2.7	4.0 ± 2.4

## Data Availability

The data that support the findings of this study are available from the corresponding author upon reasonable request.

## Abbreviations

The following abbreviations are used in this manuscript:

SUI	Stress urinary incontinence
GSM	Genito-urinary syndrome of menopause
PFMs	Pelvic floor muscles
POP	Prolapse of pelvic organs
VAS	Visual Analog Scale
